# Laparoscopic Live Donor Nephrectomy: An Initial Moroccan Experience

**DOI:** 10.7759/cureus.70713

**Published:** 2024-10-02

**Authors:** Salim Lachkar, Imad Boualaoui, Ahmed Ibrahimi, Hachem El Sayegh, Yassine Nouini

**Affiliations:** 1 Department of Urology A, Ibn Sina University Hospital, Rabat, MAR

**Keywords:** end-stage renal disease, laparoscopic, live donor nephrectomy, minimally invasive surgery, renal transplantation

## Abstract

Introduction

Laparoscopic nephrectomy is the gold standard for kidney removal in living donors, offering advantages such as reduced pain and quicker recovery. In Morocco, where end-stage renal disease (ESRD) is a growing concern, this approach could significantly impact the demand for kidney transplants. This study evaluates the safety and efficacy of laparoscopic live donor nephrectomy in the Moroccan healthcare system.

Materials and methods

Fifteen laparoscopic nephrectomies were analyzed, focusing on donor demographics, procedure details, and outcomes. Key parameters included donor age, BMI, operative time, warm ischemia time, and blood loss. Complications and graft outcomes were also assessed.

Results

The procedure was safe and effective, even in obese donors. Donors were predominantly female (80%), with an average age of 49.4 years. Obese donors had longer operative times (282 minutes vs. 220 minutes). Left kidney retrieval was preferred (95%). Warm ischemia averaged 6.27 minutes and blood loss was 207 mL. One donor had elevated creatinine postoperatively, while most maintained stable renal function. Eighteen complications, mostly minor, were reported.

Conclusion

Laparoscopic live donor nephrectomy is a safe and adaptable procedure in Morocco, offering low complication rates and favorable outcomes. It is effective for a diverse donor population, including older and obese individuals, and may help address the country's growing transplant needs.

## Introduction

Chronic kidney disease in its end stage is an escalating global public health concern, with significant implications for patients and healthcare systems worldwide. For an extended period, the conventional open-surgical approach was the primary method for renal sampling and transplantation. However, a pivotal moment in nephrology and urology occurred in 1995 when Ratner and colleagues conducted the first laparoscopic renal sampling from a living donor, initiating a transformative era in renal surgery [1].

The widespread adoption of laparoscopy in renal procedures has revolutionized the field, offering numerous advantages over traditional open surgery. Laparoscopy dramatically reduces postoperative morbidity, minimizes pain, and enhances cosmetic outcomes without compromising graft quality, vital for successful kidney transplantation [2]. Amid a persistent shortage of kidney grafts, laparoscopic renal sampling has become an asset in encouraging more living donors. By mitigating discomfort and shortening recovery, laparoscopy motivates potential donors, expanding the pool of available organs for transplantation [3]. Currently, laparoscopic renal sampling is the gold standard for kidney procurement. Its broad acceptance and implementation are evident in statistics. In 2005, around 83% of renal samplings in the United States were laparoscopic, surging to 95% by 2012 [4].

In our medical center, the conventional open surgical approach had long been the prevailing method for renal sampling procedures. However, a significant milestone in our department's history was achieved in 2017 when we performed the inaugural laparoscopic renal sampling in Morocco. This landmark event marked a pioneering achievement in our country's medical landscape and signified the commencement of a new era in renal surgery. Since that pivotal moment, we have continually refined and expanded our laparoscopic renal sampling program. In this study, we provide a comprehensive report on our initial experience, detailing our journey after conducting the first 15 cases in our series. This represents a significant stride in our pursuit of advancing the field of renal surgery and offering valuable insights into the outcomes and advantages of laparoscopic renal sampling.

## Materials and methods

The benefits of laparoscopic live donor nephrectomy compared to open surgery and other minimally invasive approaches are well-documented in the literature. In contrast to the traditional open nephrectomy, which requires a larger incision and is associated with increased postoperative pain, longer hospital stays, and delayed recovery, the laparoscopic approach offers distinct advantages [2]. These include smaller incisions, reduced blood loss, shorter warm ischemia time, and quicker recovery, allowing donors to resume normal activities sooner [3]. In addition, the transperitoneal approach employed in our study ensures better visualization of the renal anatomy, facilitating safer dissection [4]. When compared to hand-assisted or robotic-assisted nephrectomy, laparoscopic surgery provides similar clinical outcomes but with the added benefit of being more cost-effective, particularly in resource-limited settings [2].

This study was a retrospective descriptive analysis of the first 15 laparoscopic living donor nephrectomies in Ibn Sina University Hospital, located in Rabat, Morocco, which also represented the first such procedures performed in the country. During this study, we conducted these 15 laparoscopic living donor nephrectomies, and all 15 harvested kidneys were subsequently transplanted. The surgical technique for laparoscopy remained consistent throughout the study. We exclusively employed a transperitoneal approach with three to four trocars and an additional iliac incision for graft retrieval, ensuring a standardized method across all 15 cases. For ligating the renal pedicle, we utilized non-absorbable polymer Hem-o-lok® vascular clips (Weck Closure Systems, Research Triangle Park, NC, United States) for economic reasons.

All nephrectomies were carried out by a surgical team consisting of two highly experienced laparoscopic surgeons (E.S.H. and N.Y.). Both surgeons had extensive prior experience in open nephrectomy procedures, and their collective expertise in laparoscopic techniques ensured the quality and safety of each living donor nephrectomy.

Donor and recipient medical records were meticulously reviewed to compile the following data. Operative time was defined as the duration from the initial skin incision to the application of the wound bandage. Incision length consisted of the measurement and recording of the length of the extraction incision for each procedure. Blood loss represented the estimations of blood loss during the nephrectomy documented by each participating surgeon. Warm ischemia time (WIT) was the time interval between the occlusion of the renal artery and the immersion of the harvested kidney in ice slush. Delayed graft function (DGF) was defined as the need for dialysis within one week following kidney transplantation. Slow graft function (SGF) recovery was identified when the recipient's serum creatinine level remained at or above 3.0 mg% for one week post-transplantation.

All data collected during this study were presented as mean ± standard deviation. Missing data were addressed through multiple imputations, a statistically rigorous method. Imputed datasets were created to account for missing values, and analyses were conducted on these datasets. Efforts to mitigate bias included a standardized surgical approach, experienced surgeons, and meticulous data collection, enhancing internal validity.

This study was reviewed and approved by the Ethics Committee of Ibn Sina University Hospital (dated February 18, 2024), ensuring compliance with all ethical guidelines and standards for research involving human subjects.

## Results

Preoperative characteristics of donors

In our series of 15 laparoscopic renal transplant cases, donors averaged 49.4 years (±9.7), with nine being older than 50. Females predominated (12 out of 15), resulting in a male-to-female ratio of 0.25. All donors were closely related to recipients, adhering to legal requirements. The average BMI was 26 (±4.7), with four donors classified as obese (BMI 33-35), and they had received specialized nutritional support. No significant medical history was found. Common issues included hypertension, drug allergies, and prior surgeries (two cases). Hemoglobin averaged 13.31 g/dL (±1.36), and creatinine averaged 6.74 mg/L (±1.57). Vascular imaging revealed a classic pattern of one renal vein and artery in thirteen donors, while two displayed variations, including two renal veins or arteries. In addition, we noted a low left renal vein termination and an early left renal artery division, which led to the left kidney being harvested in these distinctive cases (Table 1).

**Table 1:** Perioperative donor parameters

	Mean	Standard deviation (SD)
Preoperative parameters		
Age (years)	49.4	9.7
Male-to-female ratio	0.25	-
BMI (kg/m²)	26	4.7
Hemoglobin (g/dL)	13.31	1.36
Creatinine (mg/L)	6.74	1.57
Operative parameters		
Left nephrectomy (number)	15	0
Surgery duration (minutes): Overall	245.57	105.5
Surgery duration (minutes): BMI ≤ 33	220	51.3
Surgery duration (minutes): BMI ≥ 33	282	54.9
Warm ischemia time (seconds)	376.2	40
Blood loss (mL)	207	180
Incision length (cm)	6.8	1.2
Number of renal arteries	1.07	0.25
Number of renal veins	1.07	0.25
Postoperative parameters		
Hospital stay (days)	3.2	1.5
Passed flatus (days)	1.8	0.8
Progressed to regular diets (days)	2	0.86
Follow-up (months)	35.9	22.5
Hemoglobin (g/dL)	13	2.4
Creatinine (mg/L)	10	2

Operative characteristics of donors

The left kidney was consistently used as the graft source. The average surgery duration was 245.57 minutes (±105.5), with an extension in surgery times (averaging 282 ± 54.9 minutes) observed when the donor's BMI exceeded 33, in contrast to 220 (±51.3) minutes for BMIs below 33. Warm ischemia time averaged 376.2 seconds (±40), and intraoperative blood loss averaged 207 mL (±180).

During surgery, we encountered various anatomical findings. Eight cases displayed typical renal anatomy, while seven presented atypical variations, including three previously undiagnosed anomalies. These new findings included a substantial renal-azygo-lumbar trunk, which extended surgery duration by 60 minutes; bifurcation of the left renal vein extending to its confluence with the inferior vena cava; and a notably short ureter necessitating a uretero-ureteral termino-lateral anastomosis.

Furthermore, in four cases, preoperative diagnoses concurred with intraoperative findings. Importantly, no conversions from laparoscopic to open nephrectomy occurred. The incision length was 6.8 (±1.2) cm. Major complications, defined as events posing significant risks to the donors, were not encountered. However, minor complications did arise and were either addressed laparoscopically or considered not to represent significant risks to the donors. Minor complications included splenic lacerations (one case), pneumothorax (one case), intubation/extubation difficulties (two cases), controllable vascular injuries (involving the adrenal vein and lumbar vein) (two cases), challenging manual extractions (one case), and cardiac arrhythmia (one case).

Throughout the series, graft extraction sites remained consistent in the inguinal region, with a uniform incision length (Tables 1, 2).

**Table 2:** Perioperative complications and grading according to the Clavien-Dindo classification modified by Kocak

	Description	Number	Grade	Total
Intraoperative	Splenic lacerations	1	-	8
	Pneumothorax	1	-	
	Intubation/extubation difficulties	2	-	
	Controllable vascular injuries	2	-	
	Challenging manual extractions	1	-	
	Cardiac arrhythmia	1	-	
Postoperative	Pyelonephritis	1	II a	9
	Pneumonia	2	II a	
	Erysipelas	1	I	
	Parietal infections	2	II a	
	Severe parietal infections	1	II b	
	Asymptomatic bacteriuria	1	I	
	Abdominal pain	1	I	

Postoperative donor parameters

The average hospital stay for donors was 3.2 (±1.5) days. We implemented a vital multimodal analgesia protocol to manage pain effectively, contributing to a smoother recovery process. Our analgesic regimen included paracetamol, tramadol, and nefopam as needed, with no morphine. We believe that this approach helped reduce convalescence time, lowered the risk of renal pedicle clip displacement, and mitigated complications related to autonomic nervous system activation, insulin resistance, and pro-inflammatory cytokine secretion. Donors resumed clear liquid diets in the first 24 hours, passed flatus at 1.8 (±0.82) days, and progressed to regular diets at two (±0.85) days. Importantly, the exclusively transperitoneal approach did not lead to any postoperative transit delays or complications. Our donors underwent postoperative follow-up for an average of 35.9 (±22.5) months. Hemoglobin levels averaged 13 (±2.4) g/dL. Meanwhile, the mean creatinine level was 10 (±2) mg/L. The majority of donors maintained consistently normal creatinine levels during follow-up. However, one donor exhibited a persistent increase in creatinine levels after one year (Table 1).

Throughout our study, we observed a total of nine postoperative complications, including pyelonephritis, pneumonia, erysipelas, parietal infections, severe parietal infections with clinical and biological sepsis, asymptomatic bacteriuria, and abdominal pain. These complications were effectively managed. Notably, three donors experienced no complications throughout the entire follow-up period. Our complications were categorized according to the Clavien-Dindo classification modified by Kocak, resulting in three grade I complications, five grade IIa complications, and one grade IIb complication (Table 2).

Recipient graft outcome

Fifteen kidney transplants were successfully completed. Recipient graft function, measured by post-transplant creatinine levels, averaged 1.6 (±0.5) mg/dL throughout the follow-up period. Delayed graft function, characterized as a primary nonfunction of the renal allograft, occurred once due to graft rejection. Recipient ureteral complications arose once, possibly stemming from early harvesting techniques, and were effectively treated with ureteral reimplantation.

## Discussion

In our study, the majority of donors were female (12 out of 15), with an average age of 49.4 years, consistent with the general trend seen in other studies where women are more frequently living donors. Furthermore, nine of the donors were over 50 years old. Although the use of older living donors can raise ethical questions due to the higher prevalence of chronic conditions and age-related physiological changes, many findings suggest that there is generally no correlation between the donor's age and graft survival or glomerular filtration rate decline [5]. Notably, age over 60 is not necessarily a contraindication for kidney donation, provided rigorous medical evaluation is conducted [6].

The average procedure duration was 245.57 minutes, with longer operative times observed in obese donors (BMI > 33). This observation aligns with previous data indicating that obesity can prolong surgery. Large series of laparoscopic renal donations have reported a significant percentage of obese donors with a BMI > 30, up to 24% in 1200 cases [7]. Donor BMI is a crucial consideration, as obesity can pose technical challenges during surgery. Interestingly, perioperative outcomes do not appear to significantly differ compared to donors with a BMI < 30 in large studies, particularly in terms of postoperative renal outcomes for obese donors [8]. These findings suggest that a high BMI should not be considered an absolute contraindication for kidney donation after careful case-by-case selection in high-volume centers [9].

Typically, the left kidney is favored due to its easier accessibility, resulting in retrieval rates of over 95% for the left kidney in most studies [10]. While right kidney retrieval is more technically demanding, often necessitating an additional trocar to manage the inferior vena cava and liver, as well as dealing with a shorter right renal vein, it is reserved for cases of necessity. Graft outcomes are similar between right and left kidney retrievals in larger series [11].

We found an average warm ischemia time of 6.27 minutes, consistent with similar studies reporting times between 1.95 and seven minutes [9]. Although concerns have been raised about potential graft damage due to longer warm ischemia times, the evidence suggests that even extended warm ischemia times, up to 17 minutes, do not appear to adversely affect graft survival or function [12]. Therefore, we support the notion that there is no need for undue haste in the retrieval process.

Intraoperative blood loss in our study averaged 207 mL, consistent with findings from other studies. Most reports indicate minimal blood loss, typically less than 200 mL [13]. Intraoperative blood loss is a predictive factor for postoperative complications, with a 0.2% increased risk of complications for every milliliter of blood loss [14]. Several factors, including female gender, surgeon's lack of experience, and early renal artery division, have been statistically associated with higher blood loss. Notably, the use of Hem-o-lok® clips has been shown to significantly reduce blood loss [15].

In our series, only one donor exhibited a persistent increase in creatinine levels after one year. It is important to note that our study had a relatively small sample size and larger studies have reported cases of long-term renal insufficiency in donors, with an incidence ranging from 6% to 26%. Typically, this mild renal insufficiency corresponds to KDOQI stage II and is more likely to occur in donors with higher preoperative creatinine levels or risk factors such as hypertension, obesity, or dyslipidemia [16].

Concerning pneumoperitoneum during laparoscopic kidney retrieval, it can impact renal function temporarily by reducing blood flow due to factors like CO_2_ absorption, sympathetic activity, compression, and hormonal changes [17]. However, these effects are generally reversible and not clinically significant for donors. While recipient graft function concerns exist, especially with high-pressure pneumoperitoneum, Doppler ultrasonography can assess renal function. Nevertheless, pneumoperitoneum does not significantly affect early graft function or donor renal function [18]. Low-pressure pneumoperitoneum may offer post-operative comfort without affecting renal outcomes. Measures like exsufflation, papaverine use, and ensuring urine flow can help mitigate these effects, although their utilization varies by center. Papaverine, derived from poppy latex, counteracts vasospasm during laparoscopic kidney retrieval, improving renal blood flow, diuresis, and creatinine clearance. Its application varies, with some using it routinely and others selectively based on conditions [19].

In our study, a total of 17 complications occurred. It is noteworthy that all nine postoperative complications in donors were successfully managed, primarily of low severity (Clavien-Dindo grade I). Hu et al. have shown that the presence of three or more renal arteries or a late renal vein confluence is statistically linked to postoperative complications [15]. A randomized controlled trial by Nicholson et al. demonstrated that the most frequent postoperative complications, in descending order, are hemorrhage, visceral injury, paralytic ileus, atelectasis, wound infection, and pneumonia. Some studies report a majority of grade II complications (55% to 96%), while grade III complications are rare (3.5% to 10%). Cases of grade IV complications are seldom reported in laparoscopic renal donations [20].

Our transition from 20 years of experience with open-living donor nephrectomy to laparoscopic nephrectomy presented numerous logistical and procedural challenges. Acquiring specialized laparoscopic towers, high-definition cameras, and advanced surgical instruments required significant investment and careful planning to integrate this equipment into our existing infrastructure. 

Training was a critical aspect of the transition, as laparoscopic surgery demands distinct skills compared to open nephrectomy. Our surgeons underwent extensive hands-on workshops, simulation training, and mentoring from experts to adapt to these techniques. The initial learning curve, especially for complex anatomical cases, was steep. However, over time, the team built confidence and proficiency, resulting in enhanced surgical precision and efficiency.

Moreover, our operating room staff needed to adapt to new workflows, including protocols for patient positioning, equipment management, anesthesia adjustments, and changes in postoperative care due to the different recovery trajectories associated with open and laparoscopic approaches. Comprehensive retraining ensured that the team was well-prepared for these new requirements.

Initial resistance to the transition was another challenge, particularly from staff accustomed to open surgery. Concerns about longer operative times, potential complications, and mastering new techniques were addressed through ongoing discussions, outcome reviews, and data from other centers that demonstrated the benefits of laparoscopy. Gradually, these concerns were alleviated as the advantages became clear. Despite these challenges, the transition to laparoscopic nephrectomy has transformed donor care and elevated the surgical standard at our center.

This shift to laparoscopic nephrectomy has allowed us to offer our donors significantly improved care, notably a reduction in postoperative pain and recovery time, enabling donors to resume their daily activities much faster compared to open surgery. In addition, the smaller incisions associated with laparoscopy not only provide better aesthetic outcomes but also reduce the risk of wound-related complications, greatly enhancing donor satisfaction [18].

From a technical perspective, laparoscopy has improved visualization, significantly enhancing the precision of dissections, particularly in cases involving complex vascular anatomy. In our experience, managing vascular anomalies, such as multiple renal arteries or short renal veins, has been greatly facilitated by the laparoscopic approach, thereby reducing intraoperative risks. The systematic use of thorough preoperative imaging and meticulous surgical planning has enabled us to anticipate and better manage these delicate situations.

To enhance the quality of graft retrieval, several strategies have been implemented at our center. We have focused on minimizing warm ischemia times through optimized surgical coordination and the use of advanced laparoscopic tools to expedite vessel dissection and clamping. In addition, we have adopted improved anesthetic protocols that reduce hemodynamic fluctuations during the procedure, further supporting graft preservation. The introduction of Hem-o-lok® clips for vascular ligation has proven particularly effective in reducing intraoperative blood loss, improving both donor safety and procedural efficiency. Our experience has also highlighted the benefits of using papaverine during the procedure, particularly in cases with higher ischemic risk. This vasodilator has improved renal blood flow, optimizing urine output and creatinine clearance, thereby enhancing renal function after graft retrieval. Coupled with low-pressure pneumoperitoneum, this approach ensures optimal donor comfort without compromising renal outcomes.

In recent years, technological advancements in surgery have evolved rapidly, particularly with the integration of the Internet of Things (IoT) into surgical practice. The IoT, also referred to as the Internet of Surgical Things (IoST), has the potential to revolutionize procedures such as donor nephrectomy by improving surgical precision and patient outcomes. IoT applications in telesurgery and telementoring have been demonstrated to reduce surgical hours, increase accessibility to high-quality care, and enhance surgical education. These technologies allow for real-time data sharing and remote guidance, which can be particularly beneficial in complex laparoscopic procedures like live donor nephrectomies, where precision is crucial to both donor safety and graft viability. Moreover, the use of IoT in telemonitoring post-surgical recovery can help in the early detection of complications, thus improving overall patient outcomes [21]. While the incorporation of IoT into surgical practice holds promise, further systematic research is needed to fully understand its benefits and optimize its use in the field of nephrectomy.

This study has several limitations that should be considered. First, the limited sample size may impact the generalizability of the findings, as a small number of participants might not fully represent the broader population. Second, the study was conducted at a single center, which may restrict the external validity and applicability of the results to other settings or institutions. Finally, the follow-up duration of the study may not be sufficient to capture long-term outcomes, potentially overlooking the effects that could emerge over a more extended period.

## Conclusions

Our study underscores the safety and efficacy of laparoscopic live donor nephrectomy in Morocco, demonstrating a smooth transition from traditional open surgery to minimally invasive techniques. The positive outcomes observed suggest that laparoscopic nephrectomy is not only feasible but also advantageous in our context, potentially revolutionizing kidney transplant practices and addressing the pressing public health issue of end-stage renal disease. This advancement could significantly improve transplant outcomes and patient quality of life. 

While these preliminary results are promising, further research is warranted to explore the long-term benefits and to refine the technique. Continued investigation and expansion of this approach will be crucial for optimizing kidney transplantation practices and meeting the growing demand for renal replacements. Overall, the study instills optimism and highlights the potential for a transformative impact on the future of kidney transplantation.
